# Evolutionary divergence of the vertebrate *TNFAIP8* gene family: Applying the spotted gar orthology bridge to understand ohnolog loss in teleosts

**DOI:** 10.1371/journal.pone.0179517

**Published:** 2017-06-28

**Authors:** Con Sullivan, Christopher R. Lage, Jeffrey A. Yoder, John H. Postlethwait, Carol H. Kim

**Affiliations:** 1 Department of Molecular and Biomedical Sciences, University of Maine, Orono, Maine, United States of America; 2 Graduate School of Biomedical Sciences and Engineering, University of Maine, Orono, Maine, United States of America; 3 Program in Biology, University of Maine - Augusta, Augusta, Maine, United States of America; 4 Department of Molecular Biomedical Sciences, North Carolina State University, Raleigh, North Carolina, United States of America; 5 Institute of Neuroscience, University of Oregon, Eugene, Oregon, United States of America; INRA, FRANCE

## Abstract

Comparative functional genomic studies require the proper identification of gene orthologs to properly exploit animal biomedical research models. To identify gene orthologs, comprehensive, conserved gene synteny analyses are necessary to unwind gene histories that are convoluted by two rounds of early vertebrate genome duplication, and in the case of the teleosts, a third round, the teleost genome duplication (TGD). Recently, the genome of the spotted gar, a holostean outgroup to the teleosts that did not undergo this third genome duplication, was sequenced and applied as an orthology bridge to facilitate the identification of teleost orthologs to human genes and to enhance the power of teleosts as biomedical models. In this study, we apply the spotted gar orthology bridge to help describe the gene history of the vertebrate *TNFAIP8* family. Members of the *TNFAIP8* gene family have been linked to regulation of immune function and homeostasis and the development of multiple cancer types. Through a conserved gene synteny analysis, we identified zebrafish orthologs to human *TNFAIP8L1* and *TNFAIP8L3* genes and two co-orthologs to human *TNFAIP8L2*, but failed to identify an ortholog to human *TNFAIP8*. Through the application of the orthology bridge, we determined that teleost orthologs to human *TNFAIP8* genes were likely lost in a genome inversion event after their divergence from their common ancestor with spotted gar. These findings demonstrate the value of this enhanced approach to gene history analysis and support the development of teleost models to study complex questions related to an array of biomedical issues, including immunity and cancer.

## Introduction

The *tumor necrosis factor-alpha-induced protein 8* (*TNFAIP8*) gene family has recently come to prominence as a regulator of several physiological and pathological processes, in particular with relation to immunity and cancer [1]. The *TNFAIP8* gene, for which the gene family was named, was originally identified in a differential display screen of head and neck squamous cell carcinoma cell lines [2]. The *TNFAIP8* gene was subsequently shown to be an early responder to TNF-alpha stimulation in human umbilical vein endothelial cells [3] and to be expressed in variety of normal tissues and cancer cell lines [4]. The human *TNFAIP8* gene family consists of four genes: *TNFAIP8* (located in chromosome 5q23.1), *TNFAIP8L1* (19p13.3), *TNFAIP8L2* (1q21.3), and *TNFAIP8L3* (15q21.2) [5, 6]. The proteins encoded by members of this gene family are unique in structure. Each possess seven alpha helices that surround a hydrophobic core thought to play a significant role in lipid second messenger signaling [7, 8]. The *TNFAIP8* and *TNFAIP8L2* genes participate in immunity and inflammation [6, 9–12], while all members of the *TNFAIP8* gene family have been associated with cancers of various types, including those affecting the stomach [13–18], liver [11, 17, 19–22], prostate [23], lung [7, 24, 25], esophagus [7, 24, 25], and cervix [7, 26]. Although the *TNFAIP8* gene family has been associated with inflammation, immunity, and cancer, little is known about the mechanisms by which these genes function and the evolutionary origins of the family are not yet fully understood.

Genetically-tractable teleost fish models like zebrafish, medaka, and platyfish have become indispensable tools in biomedicine that can be used to understand the function of gene families like *TNFAIP8* [27]. Several large-scale genomic events, however, can hinder the identification of teleost orthologs of biomedically relevant genes; these events include two rounds of early vertebrate genome duplication (VGD1 and VGD2), lineage-specific loss of various ohnologs (gene paralogs derived from genome duplication), the teleost genome duplication (TGD), and subsequent rapid sequence divergence [28]. Recently, the genome of the spotted gar, a representative of the Holostei (sister lineage to teleosts, which did not undergo the TGD), was sequenced and found to provide an orthology bridge between teleost and human genomes, facilitating the identification of zebrafish orthologs to human genes [28]. In the current study, we traced the gene history of the vertebrate *TNFAIP8* gene family. We establish the human *TNFAIP8*, *TNFAIP8L1*, *TNFAIP8L2*, and *TNFAIP8L3* genes as paralogs based on conserved synteny. We identify zebrafish orthologs to *TNFAIP8L1* and *TNFAIP8L3* and two co-orthologs to *TNFAIP8L2* (termed *tnfaip8l2a* and *tnfaip8l2b*) through conserved synteny, and we determine that an ortholog to the human *TNFAIP8* gene was lost during teleost evolution. Through application of the spotted gar orthology bridge, we show that zebrafish and stickleback *tnfaip8* were likely lost in a genome inversion event that occurred in the teleost lineage after it diverged from the spotted gar lineage. Phylogenetic analysis of the TNFAIP8 family with representative protein sequences from mammals, diapsids (birds and other ‘reptiles’), amphibians, and fish support our conclusions from the gene history analysis. A clearer understanding of vertebrate gene histories like the *TNFAIP8* family will provide better-informed applications of the zebrafish model system to study biological questions related to human and animal health and disease.

## Materials and methods

### Nomenclature conventions

Nomenclature rules for vertebrate genes and proteins follow accepted conventions. This work presents gene and protein nomenclature for specific species according to their respective naming conventions (e.g. for common name (*species*), *gene*, protein, we use: zebrafish (*Danio rerio*), *tnfaip8l1*, Tnfaip8l1 [https://wiki.zfin.org/display/general/ZFIN+Zebrafish+Nomenclature+Guidelines]; mouse (*Mus musculus*), *Tnfaip8l1*, TNFAIP8L1 [http://www.informatics.jax.org/mgihome/nomen/gene.shtml]; human (*Homo sapiens*), *TNFAIP8L1*, TNFAIP8L1 [http://www.genenames.org/]; frog (*Xenopus tropicalis*), *tnfaip8l1*, Tnfaip8l1 [http://www.xenbase.org/gene/static/geneNomenclature.jsp]; chicken (*Gallus gallus*) and turkey (*Meleagris gallopavo*), *TNFAIP8L1*, TNFAIP8L1 [http://birdgenenames.org/cgnc/guidelines] and *Drosophila melanogaster*, *CG4091*, CG4091 [http://flybase.org/static_pages/docs/nomenclature/nomenclature3.html]. Many organisms lack formalized gene and protein nomenclature conventions. We apply zebrafish nomenclature conventions to stickleback [*Gasterosteus aculeatus*], spotted gar [*Lepisosteus oculatus*], and coelacanth [*Latimeria chalumnae*] and human nomenclature conventions to Chinese softshell turtle (*Pelodiscus sinensis*) and anole lizard (*Anolis carolinensis*) genes and proteins.

### Comparative genomics

Conserved synteny analyses were performed using the Synteny Database [29]. Amino acid sequence alignments were generated using standard procedures in Clustal Omega [30] available at http://www.ebi.ac.uk/Tools/msa/clustalo/. Molecular phylogenetic analyses were performed using the PHYLIP (phylogeny inference package) software version 3.6b (distributed by the author J. Felsenstein, Department of Genome Sciences, University of Washington, Seattle at http://evolution.gs.washington.edu/phylip.html) [31]. Amino acid sequences were bootstrapped 1000 times using the program SEQBOOT. Bootstrapped amino acid sequences were used to compute distance matrices under the Jones-Taylor-Thornton (JTT) model of amino acid replacement (PROTDIST). A phylogenetic tree was generated from each distance matrix with the Neighbor-Joining (NJ) method, (NEIGHBOR) [32] and an extended majority rule consensus tree was generated [33] from this set of phylogenetic trees (CONSENSE). The consensus tree consists of monophyletic groups with percentages indicating how often each group occurred in the bootstrapped data.

## Results

### The *TNFAIP8* gene family originated in two rounds of vertebrate genome duplication (VGD)

**Fig 1:**
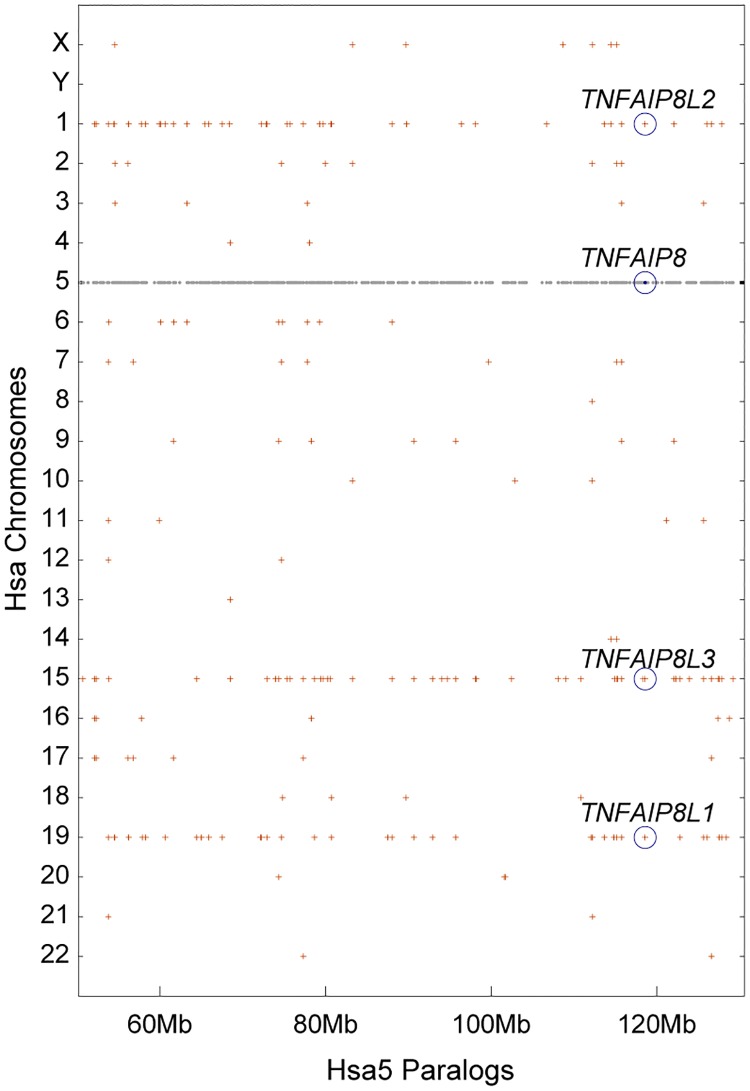
Genomic distribution of human *TNFAIP8*-family genes. Grey dots along Hsa5 represent genes whose paralogs are plotted directly above or below the dot on the human chromosomes on which they occur. Note that chromosomes 1, 15, and 19 share many paralogs with the *TNFAIP8*-containing region of Hsa5. This result suggests that these four genes originated as ohnologs in rounds 1 and 2 of the vertebrate genome duplication events (VGD1 and VGD2).

To investigate the evolutionary origins of the four human *TNFAIP8* family genes (*TNFAIP8*, *TNFAIP8L1*, *TNFAIP8L2*, and *TNFAIP8L3)*, we used the Dotplot function of the Synteny Database [29, 34] to visualize the genomic distribution of the paralogs of human chromosome 5 (Hsa5) genes on other human chromosomes. Results revealed that the region of Hsa5 between about 50 Mb and 130 Mb is paralogous to regions of Hsa1, Hsa15, and Hsa19 (Fig 1), indicating that these sequences arose by duplication of large chromosomal regions or entire chromosomes. According to Ohno’s hypothesis [35], the simplest explanation for this observation is that these four chromosome segments arose from the two rounds of whole genome duplication (WGD) that occurred at the base of the vertebrate radiation (i.e. VGD1 and VGD2) [36–38]. Therefore, we infer that the four human *TNFAIP8*-related genes are ohnologs from the VGD events. In order to determine how the genes partitioned during VGD1 and VGD2, we compared their exon-intron structures. According to Ensembl human genome assembly GRCh38.p7 (GCA_000001405.22), the human *TNFAIP8* gene encodes seven protein-coding transcript isoforms (*TNFAIP8*-001 contains 2 exons; *TNFAIP8*-002: 3 exons; *TNFAIP8*-003: 3 exons; *TNFAIP8*-004: 2 exons; *TNFAIP8*-006: 2 exons; *TNFAIP8*-007: 2 exons; and *TNFAIP8*-008: 3 exons; isoform *TNFAIP8*-005 is not represented in this assembly). The *TNFAIP8L1* gene encodes two protein coding transcripts (*TNFAIP8L1*-001 and *TNFAIP8L1*-201), which share a common ORF but differ in their 5’ UTR. The *TNFAIP8L1* gene also encodes a single exon, processed transcript that does not appear to encode a protein and is of unknown function (*TNFAIP8L1*-002). The *TNFAIP8L2* gene encodes a single transcript isoform that contains 2 exons (*TNFAIP8L2*-001). Interestingly, one of the splice forms of *SCNM1*, which is the nearest downstream neighbor of *TNFAIP8L2* and is transcribed in the same direction, shares a portion of its 5’ UTR with the 5’UTR of *TNFAIP8L2*, according to the Ensembl human genome assembly GRCH38.p10. The *TNFAIP8L3* gene encodes two protein-coding transcripts: the larger transcript (*TNFAIP8L3*-001) possesses three exons, and the smaller transcript (*TNFAIP8L3*-002) possesses two exons. Exon 2 and 3 of *TNFAIP8L3*-001 are shared by exons 1 and 2 of *TNFAIP8L3*-002. A two-exon, sense intronic transcript is encoded from this locus from intron sequence located between exons 2 and 3 of the *TNFAIP8L3*-001 transcript.

We next attempted to investigate the question of the order of evolution by examining the amino acid sequences encoded by the transcripts. Based on a Clustal Omega amino acid alignment proteins encoded by TNFAIP8 [Ensembl ENSP00000274456, transcript *TNFAIP8*-006], TNFAIP8L1 [Ensembl ENSP00000331827, transcript *TNFAIP8L1*-001], TNFAIP8L2 [Ensembl ENSP00000357906, transcript *TNFAIP8L2*-001], and TNFAIP8L3 [NCBI GenPept AAI27703.1, transcript *TNFAIP8L3*-002] display 50–57% sequence identity (S1 Fig). Each of these proteins contains a conserved TIPE2 homology (TH) domain consisting of seven alpha helices (α0–α6) [7]. TNFAIP8L3 possesses a unique N-terminus that has been associated with cell growth and survival [7]. Based on the amino acid identity and domain structure, it was not possible to infer any conclusions regarding the order of evolution for these genes.

### Zebrafish possesses orthologs to *TNFAIP8L1*, *TNFAIP8L2*, and *TNFAIP8L3*

**Fig 2:**
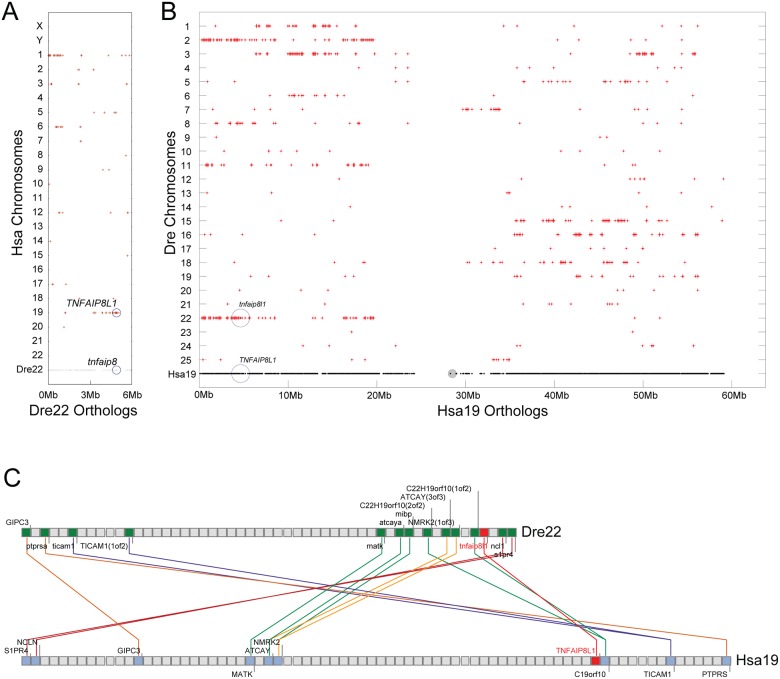
Zebrafish possesses orthologs to human *TNFAIP8L1*. (A) Grey dots along Dre22 represent genes whose orthologs are plotted on the human chromosomes on which they occur. Red dots represent regions of orthology between zebrafish and human chromosomal segments. Zebrafish (*Danio rerio*) chromosome 22 (Dre22), which contains the *tnfaip8* gene incorrectly annotated in genome assembly Zv9, shares conserved synteny with Hsa19, which contains the human *TNFAIP8L1* gene. An ortholog to human *TNFAIP8* could not be located in the zebrafish genome. (B) Grey dots along Hsa19 represent genes whose orthologs are plotted above the dot on the zebrafish (Dre) chromosomes on which they occur. Red dots represent regions of orthology between Hsa19 and each zebrafish chromosome. Between 0 and 10 Mb, Hsa19 has conserved syntenies with Dre2 and Dre22, which contains the *tnfaip8l1* ortholog. (C) Composite cluster mapping shows conserved synteny with the regions surrounding zebrafish *tnfaip8l1* (red letters) and human *TNFAIP8L1* (red letters), with several local inversions (as illustrated by the crossed lines).

In our initial investigation using Ensembl Zv9 (www.ensembl.org), we determined that the zebrafish genome encodes four members of the vertebrate *TNFAIP8* gene family, called *tnfaip8* (ENSDARG00000086457), *tnfaip8l2a* (ENSDARG00000075592), *tnfaip8l2b* (ENSDARG00000046148), and *tnfaip8l3* (ENSDARG00000088709). To investigate relationships of members of the zebrafish *tnfaip8* gene family to their human orthologs, we compared conserved syntenies of chromosome segments containing the four zebrafish genes to the human genome. We found that the gene called ‘*tnfaip8*’ (ENSDARG00000086457) occupies a chromosome segment on Dre22 whose genes are mostly orthologous to the region of Hsa19 that contains *TNFAIP8L1*, not to the region of Hsa5 that contains *TNFAIP8* (Fig 2A). This result casted doubt on the original nomenclature assignments. Furthermore, BLAST searches using either human *TNFAIP8* or *TNFAIP8L1* as queries both returned ENSDARG00000086457, a gene located on Dre22 at nucleotide position 4,857,840, as the best hit. This gene has conserved synteny with the human gene *TNFAIP8L1* (Hsa19) but not with *TNFAIP8* (Hsa5). The region of Hsa19 between 0 and 10Mb displays conserved synteny with both Dre 22 and Dre2 (Fig 2B). ENSDARG00000086457 on Dre22 is located in the middle of this region of conserved synteny (Fig 2B). The local region around ENSDARG00000086457 shows conserved synteny with the region surrounding the human gene *TNFAIP8L1*, with several local inversions (Fig 2C). We conclude that the gene ENSDARG00000086457, which was annotated as *tnfaip8* in the Zv9 assembly, instead should be *tnfaip8l1*. In the update to the Zv9 assembly, known as Genome Reference Consortium Zebrafish Build 10 (GRCz10), the locus originally designated as *tnfaip8* was changed to *tnfaip8l1*, which is in agreement with our earlier analysis (ENSDARG00000086457 22:4769086–4787454:-1). We carefully examined EST data that had been previously associated with the incorrectly annotated term “zebrafish *tnfaip8*” (located on UniGene [https://www.ncbi.nlm.nih.gov/unigene] at UGID: 2554148) and determined that there was no evidence of a zebrafish *tnfaip8* ortholog in the expression data.

**Fig 3:**
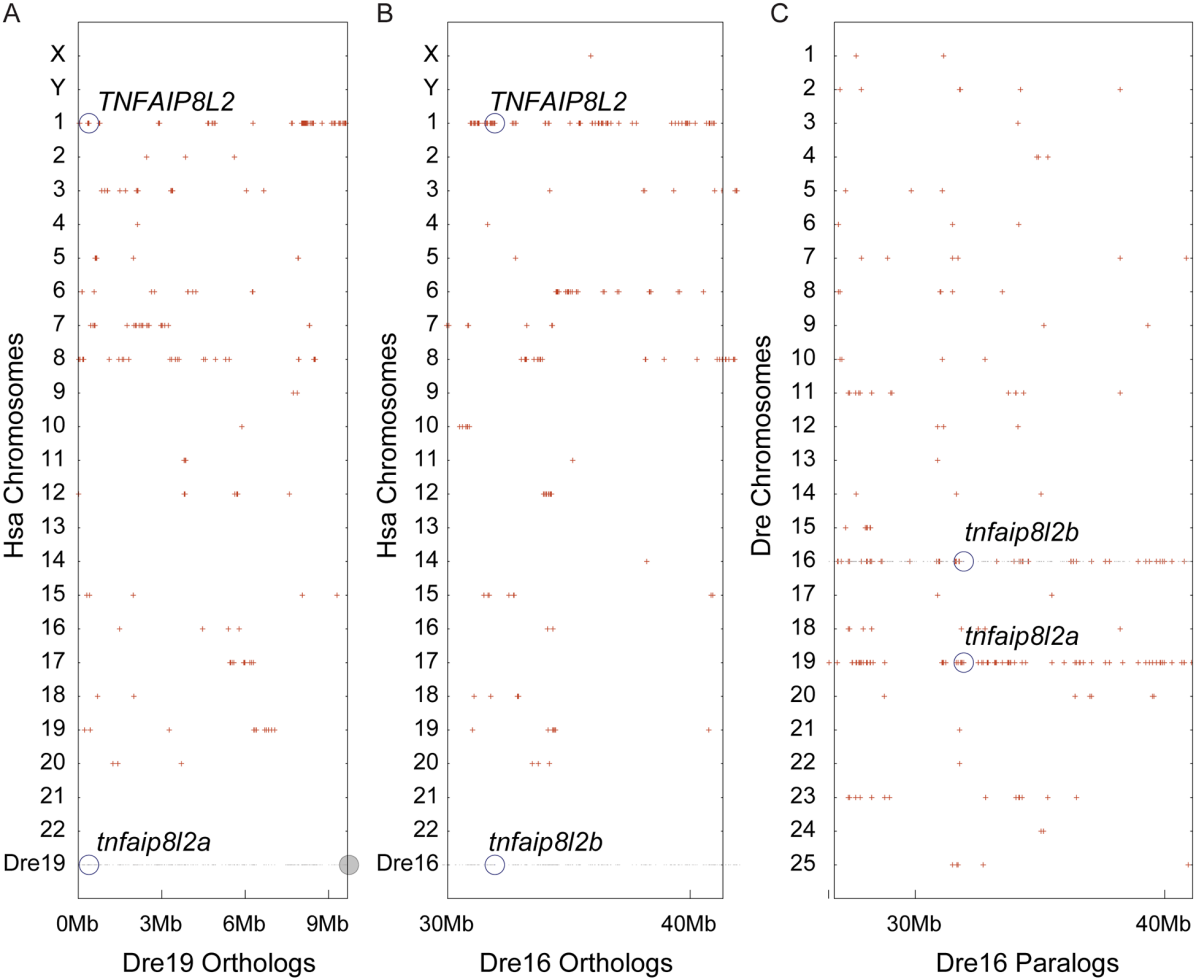
Zebrafish possesses co-orthologs to human *TNFAIP8L2*. (A) Dre19, which contains the zebrafish *tnfaip8l2a* gene, and (B) Dre16, which contains the *tnfaip8l2b* gene, each share conserved synteny with Hsa1, which contains the human *TNFAIP8L2* gene. Gray dots represent genes on Dre 19 (A) and Dre16 (B) with human (Hsa) orthologs, represented as red dots. (C) Dre16 and Dre19 show conserved synteny (red dots that are aligned) [39]; *tnfaip8l2a* and *tnfaip8l2b* are TGD co-orthologs of the human *TNFAIP8L2* gene.

Comparative genomic analyses also showed that zebrafish has two co-orthologs of human *TNFAIP8L2*, called *tnfaip8l2a* (ENSDARG00000075592) and *tnfaip8l2b*. (ENSDARG00000046148). Conserved synteny analysis verified that the sections of the zebrafish genome on chromosomes Dre19 and Dre16 that contain these two genes are both orthologous to the section of Hsa1 that contains *TNFAIP8L2*, consistent with their gene nomenclature (Fig 3A and 3B). Because Dre16 and Dre19 are predicted to be derived from a duplicated chromosome that arose in the teleost genome duplication (TGD) [39], most of Dre16 is paralogous to Dre19 (Fig 3C).

**Fig 4:**
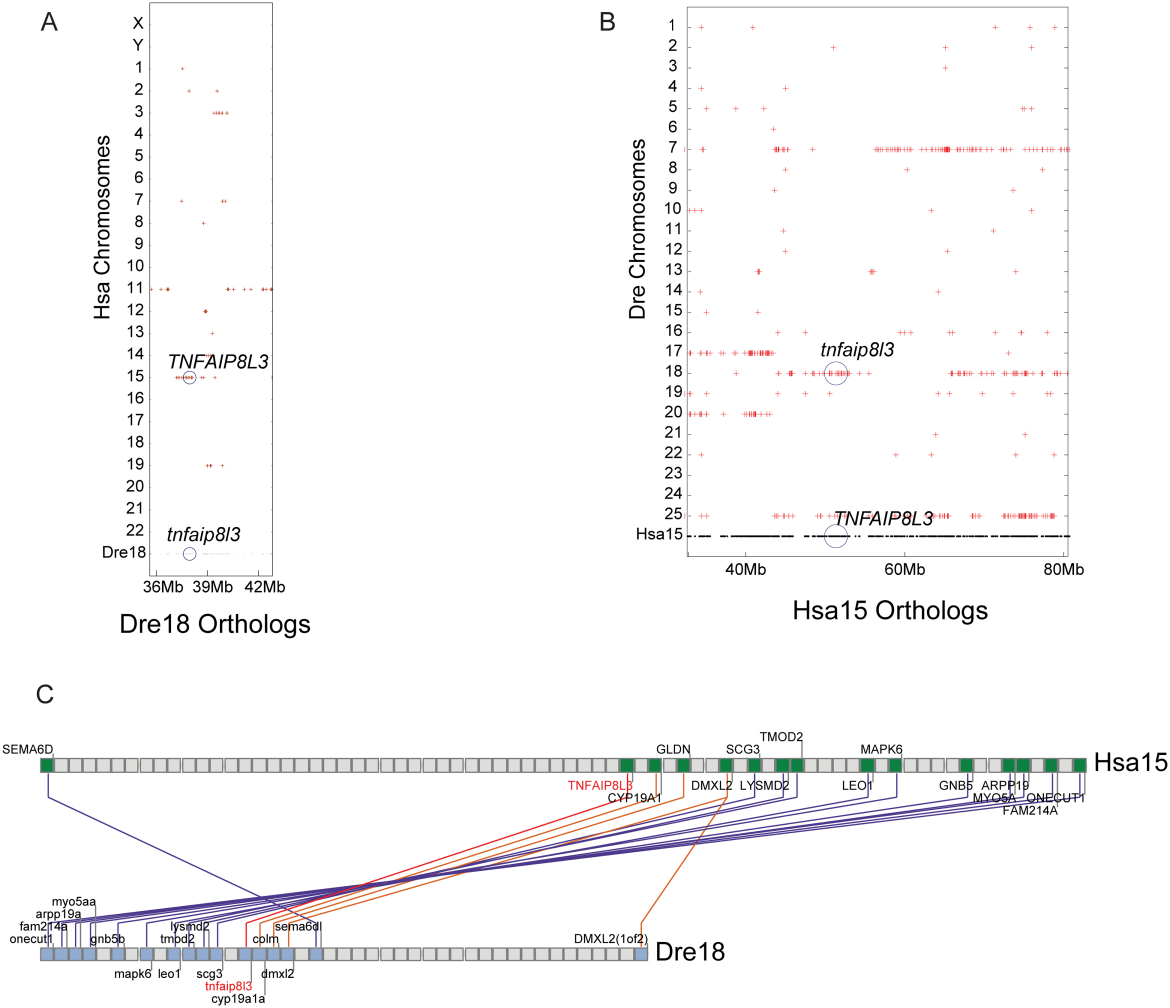
Zebrafish possesses an ortholog of human *TNFAIP8L3*. (A) Red dots represent regions of orthology between zebrafish and human chromosomal segments. Zebrafish (*Danio rerio*) chromosome 18 (Dre18) (gray dots), which contains the *tnfaip8l3* gene, shares orthology with Hsa15, which contains the human *TNFAIP8L3* gene. (B) Red dots represent regions of orthology between Hsa15 (gray dots) and the zebrafish genome. Evidence of two co-orthologous regions in zebrafish, likely arising from the TGD, are present in the region on Dre18 and Dre25. The *tnfaip8l3* gene is present on Dre18. Additional co-orthologons are present on Dre17 and Dre20 and Dre7 and Dre25. The presence of the *tnfaip8l3* orthologon on Dre18 is likely caused by a translocation event that occurred with Dre7. (C) Composite cluster mapping showing conserved synteny between Hsa15 and Dre18 in the regions surrounding human *TNFAIP8L3* (red letters) and zebrafish *tnfaip8l3* (red letters).

Analysis of conserved syntenies further verified the assignment of orthologies for zebrafish *tnfaip8l3* and human *TNFAIP8L3* (Fig 4A and 4B). The human *TNFAIP8L3* gene lies on Hsa15 in a chromosome segment that has 14 pairs of orthologs—most in the same order—with zebrafish Dre18, including *tnfaip8l3* (Fig 4C). As expected from the TGD, the region of chromosome Hsa15 that contains *TNFAIP8L3* has two co-orthologous regions in zebrafish, one on Dre18 that contains *tnfaip8l3*, and one on Dre25 (Fig 4B). The duplicated paralogons (i.e. paralogous chromosomal regions, derived by duplication from a common ancestral region) from the TGD are evident in the zebrafish co-orthologs of Hsa15 as the region between about 35 Mb and 43 Mb shows duplicated paralogons on Dre17 and Dre20, and the region from 43 Mb to 65 Mb is duplicated on Dre25 and on Dre7 with a translocation to Dre18 containing *tnfaip8l3*.

These data lead to the conclusion that zebrafish has one ortholog of *TNFAIP8L3*, two co-orthologs of *TNFAIP8L2*, and one ortholog of *TNFAIPL1* (that had been incorrectly named *tnfaip8* in the previous genome version) and that a true ortholog of human *TNFAIP8* is absent from the zebrafish genome.

### Evolutionary history of the *tnfaip8* in teleosts

**Fig 5:**
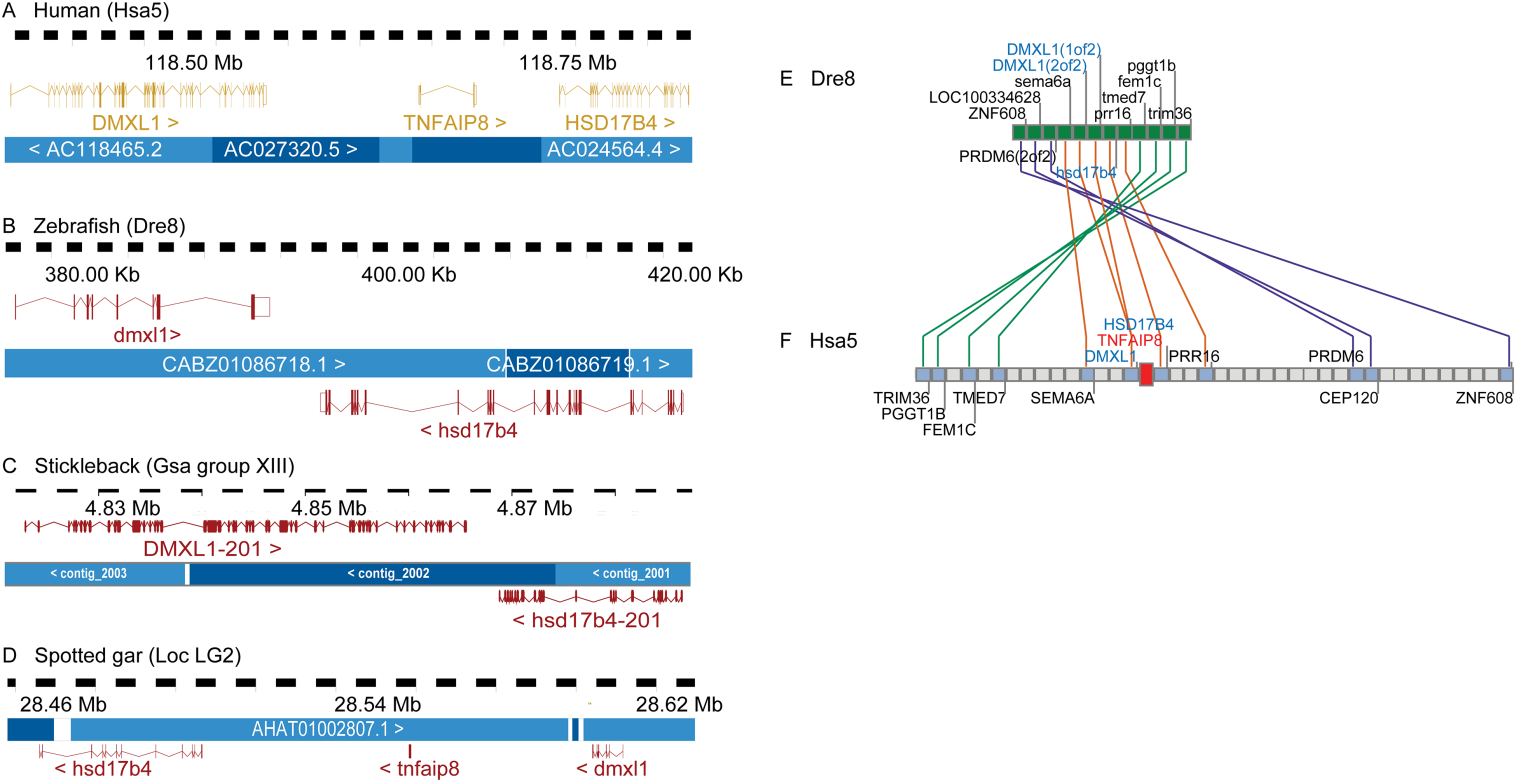
The *tnfaip8* gene was lost in a genome inversion event after the divergence of the teleost and spotted gar lineages. *TNFAIP8* is flanked by *DMXL1* and *HSD17B4* in (A) humans and rayfin fishes that diverged before the teleost genome duplication like the (D) spotted gar. The three genes are transcribed in the same direction in both human and gar genomes. In teleosts including (B) zebrafish and (C) stickleback, *tnfaip8* is missing and *dmxl1* and *hsd17b4* are transcribed in opposite directions. (E, F) Dre8 and Hsa5 share conserved synteny in the region surrounding *TNFAIP8*. Crossing lines indicate shifts in gene order consistent with a chromosome inversion event. The other breakpoint for the inversion occurred between *hsd17b4* and *prr16*, because they transcribe in opposing directions in the zebrafish (E) and in the same direction in humans (F).

In the human genome, *TNFAIP8* lies at 118.6Mb on chromosome Hsa5 and is flanked by *DMXL1* and *HSD17B4*. All three genes are transcribed in the same direction (Fig 5A). Tandem duplicate orthologs of *DMXL1* (*dmxl1*[1of2] and *dmxl1*[2of2]) and an adjacent ortholog of *HSD17B4* lie adjacent to one another, 3.2 kB apart at the left tip of Dre8 at location 0.3 Mb. The 3.2 Kb between zebrafish *dmxl1* and *hsd17b4* contains no sequence recognizable as a *TNFAIP8* ortholog (or any other known genetic element) (Fig 5B) as found in the human genome. In addition, the zebrafish *dmxl1* and *hsd17b4* genes are transcribed in opposite directions (Fig 5B) rather than in the same direction as observed in the human genome. In stickleback, a teleost distantly related to zebrafish, *dmxl1* and *hsd17b4* are also adjacent and transcribed in opposite directions as in zebrafish (Fig 5C), suggesting that this arrangement is shared broadly among teleosts and that the *tnfaip8* gene in teleosts represents an ohnolog gone missing.

To determine whether the teleost gene arrangement or the human arrangement is the ancestral condition, we inspected the genome of spotted gar, a rayfin fish like teleosts, but one that represents the latest lineage that diverged from the teleost lineage before the TGD [39–43]. The spotted gar genome encodes the *dmxl1*, *tnfaip8*, and *hsd17b4* genes in the same order and orientation as in the human genome (Fig 5D). These results indicate that the ancestral condition was *dmxl1*> *tnfaip8*> *hsd17b4>* and that the *tnfaip8* ohnolog was lost from the teleost lineage after the divergence of gar and teleost lineages. Because *dmxl1* and *hsd17b4* are oriented in opposite directions in teleosts but in the same direction in gar and human, we conclude that an inversion with a breakpoint between the *DMXL1* and *HSD17B4* genes occurred in the teleost lineage after it diverged from the gar lineage. The other breakpoint of this inversion was between *hsd17b4* and *prr16*, which are transcribed in the same direction in human but in opposite directions in zebrafish (Fig 5E and 5F). We hypothesize that the inversion breakpoint between *dmxl1* and *hsd17b4* occurred within the ancestral *tnfaip8* gene or its regulatory elements, thus destroying its activity, after which this gene likely became a pseudogene that subsequently disappeared without a trace.

To confirm that the genomic regions shown in Fig 5A and 5B represent segments that are truly orthologous, we took a broader look at the chromosome region that encodes *DMXL1*, *TNFAIP8* and *HSD17B4* using the Synteny Database [29]. A group of 12 zebrafish genes including *dmxl1* and *hsd17b4* (Fig 5E) shares conserved synteny with a region of Hsa5 that contains *TNFAIP8* (Fig 5F). At least one additional inversion event occurred in this region after the teleost and human lineages diverged, as evidenced by the series of crossing lines in Fig 5E and 5F. These conserved synteny data strongly support the conclusion that the human and zebrafish chromosome segments shown in Fig 5 are indeed orthologous. The simplest explanation of these data is that teleosts, including zebrafish, have no ortholog of the human and gar *TNFAIP8* gene at this locus because it was destroyed in an inversion event that occurred after the divergence of zebrafish and gar lineages but before the divergence of stickleback and zebrafish lineages.

### Location of the TGD paralog of the *dmx1l*, (*tnfaip8*), *hsd17b4* region in zebrafish

**Fig 6:**
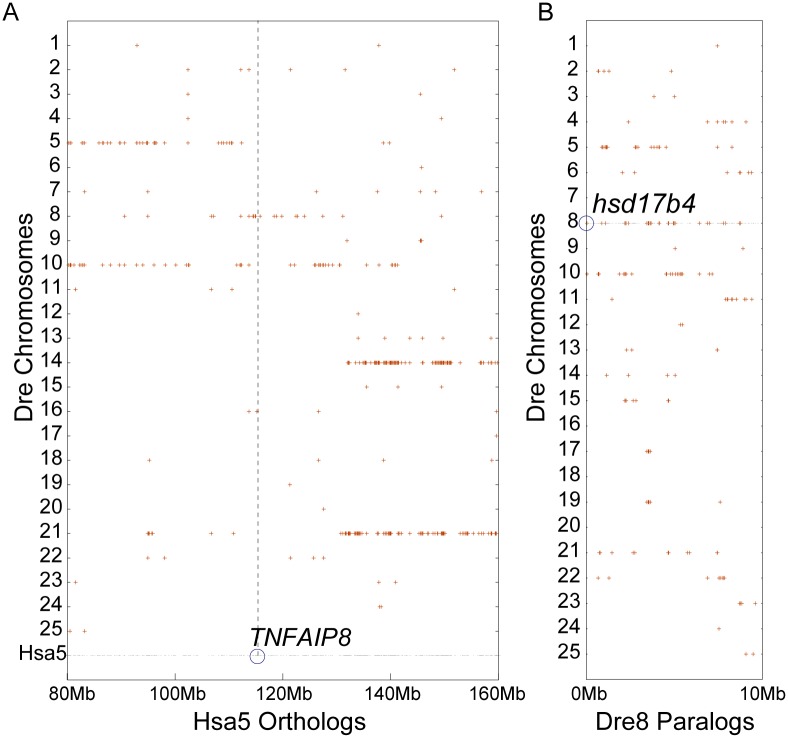
After the TGD, the chromosome region surrounding the ancestral *tnfaip8* gene reverted to a single copy. Grey dots represent regions of orthology between zebrafish chromosomes and human Hsa5. (A) The region surrounding human *TNFAIP8* has two sets of co-orthologous paralogons in the zebrafish genome on Dre5 and Dre10 (left) and Dre14 and Dre21 (right). (B) No obvious paralogons are evident in the left tip of Dre8 (containing *tnfaip8*-neighboring gene *hsd17b4*). Dre5 and Dre10 show evidence of distant homology.

**Fig 7:**
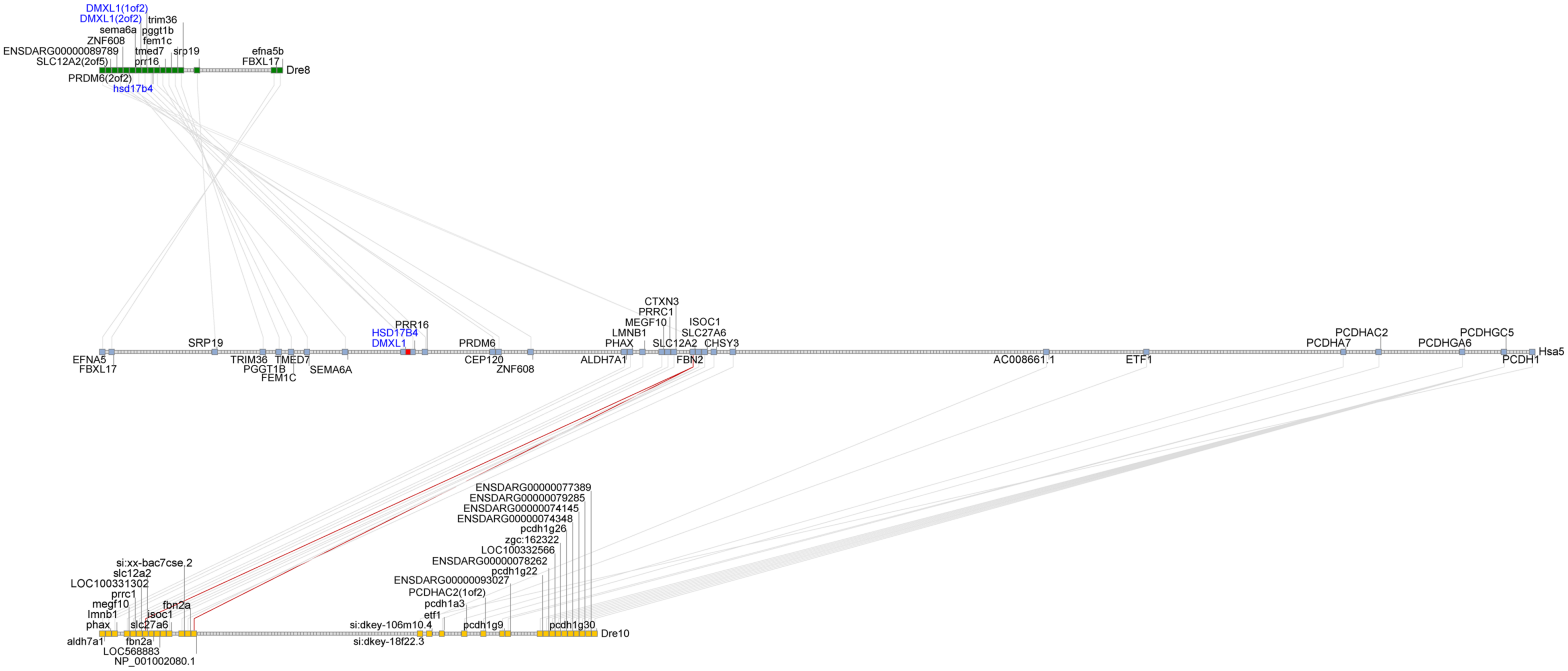
Adjacent regions of Hsa5 show evidence of orthology with Dre8 and Dre10. Only a single copy of the region lacking the *TNFAIP8* ortholog is present in the zebrafish on Dre8.

Due to the TGD, zebrafish possesses two paralogous copies of many regions of the human genome [28, 39, 41, 44, 45]. The Dotplot tool of the Synteny Database helped locate the region of the zebrafish genome that is a TGD paralog of the Dre8 region containing *dmxl1*, (*tnfaip8*), *hsd17b4*, [29]. Results revealed that the central portion of the long arm of Hsa5 from 80Mb to about 117Mb clearly has two co-orthologous paralogons on a portion of zebrafish chromosomes Dre5 and Dre10 (Fig 6A, left), and the region of Hsa5 between about 130Mb to the end of the chromosome has duplicates on Dre14 and 21 (Fig 6A, right). The *TNFAIP8* gene, however, resides in the region of Hsa5 between 117Mb and 125Mb, whose TGD paralog is less unclear. To investigate this point further, we looked for paralogs of genes occupying the left tip of Dre8 (note that *hsd17b4* is the tenth gene from the left telomere). Results failed to reveal an obvious paralogon for this region, although Dre5 and Dre10 possess some more distantly related homologs (Fig 6B). The analysis of individual genes confirmed this finding, and showed that the portion of Hsa5 containing *TNFAIP8* is orthologous to a portion of Dre8 but that the adjacent region on Hsa5 is orthologous to a part of Dre10 (Fig 7). We conclude that the zebrafish genome lacks not only an ortholog of *TNFAIP8*, but also that it possesses a single ortholog of the chromosomal region in which the spotted gar Dre8 gene is embedded. Given an absence of suitable genomic analyses in the most basally diverging teleosts, the eels and bony tongues, it is difficult given available information to determine whether the loss of *tnfaip8* occurred before or after the TGD. If *tnfaip8* loss occurred after the TGD, then one duplicated copy was likely lost in the inversion event discovered above, and the other duplicated copy was likely lost by a mechanism that deleted not only it but the surrounding genes as well.

### Phylogenetic analysis of the *TNFAIP8* family

**Fig 8:**
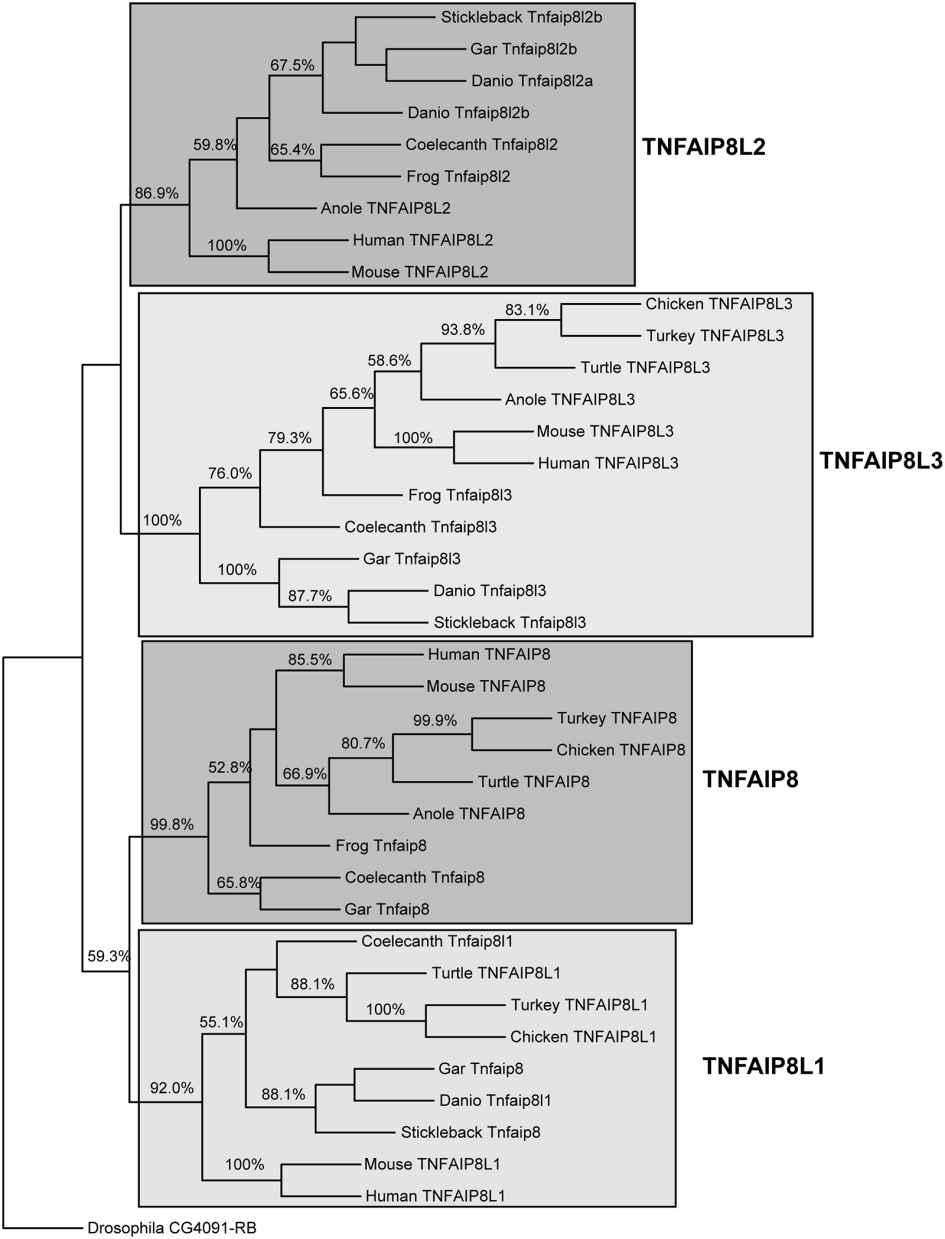
Phylogeny of the TNFAIP8 family of proteins. Sequences were collected from Ensembl (www.ensembl.org) and GenPept (http://www.ncbi.nlm.nih.gov/protein) and aligned using Clustal Omega (S2 Fig) [30]. Sequence identifiers are provided in S2 Fig. *Drosophila* sequence CG4091-RB was used as an outgroup. The unrooted consensus tree with % bootstrap support for each node (above 50%) is presented. The common and binomial names of animals comprising the alignment (S2 Fig) and cladogram are as follows: zebrafish (*Danio rerio*), mouse (*Mus musculus*), human (*Homo sapiens*), frog (*Xenopus tropicalis*), chicken (*Gallus gallus*) and turkey (*Meleagris gallopavo*), stickleback (*Gasterosteus aculeatus*), spotted gar (*Lepisosteus oculatus*), coelacanth (*Latimeria chalumnae*), Chinese softshell turtle (*Pelodiscus sinensis*) and anole lizard (*Anolis carolinensis*).

The gene history analyses described above are supported by phylogenetic analysis of 39 TNFAIP8 family proteins with representatives from fish, amphibians, reptiles, birds, and mammals, and *Drosophila* as an outgroup. All TNFAIP8 sequences (with the exception of *Drosophila*) form a monophyletic group with 99.8% support (Fig 8 and S2 Fig). All TNFAIP8L1 sequences form a monophyletic group with 92.0% support. This group includes the zebrafish Tnfaip8l1 sequence (previously named Tnfaip8), as well as the stickleback Tnfaip8 sequence and one of the gar Tnfaip8 sequences. These zebrafish, stickleback, and gar data infer similarity to Tnfaip8l1 rather than Tnfaip8 across taxa and complement gene synteny analyses. It also supports our claim that the *tnfaip8* gene has been lost in sequenced teleost genomes. Analyzed TNFAIP8L2 sequences formed a monophyletic group with 86.9% support, and TNFAIP8L3 sequences formed a monophyletic group with 100% support. Taken together, the phylogenetic analysis supports our conserved synteny analysis identifying one zebrafish ortholog to mammalian *Tnfaip81l* (*tnfaip8l1*), two zebrafish co-orthologs to mammalian *Tnfaip8l2* (*tnfaip8l2a* and *tnfaip8l2b*), and one zebrafish ortholog to mammalian *Tnfaip8l3* (*tnfaip8l3*). The phylogenetic tree suggests, although without strong support (59% for the TNFAIP8/TNFAIP8L1 clade and 48.4% for the TNFAIP8L2/TNFAIP8L3 clade), that VGD1 produced a *TNFAIP8/8L1* ancestor gene and a *TNFAIP8L2/8L3* ancestor gene, and then VGD2 produced the full group of four genes (*TNFAIP8*, *TNFAIP8L1*, *TNFAIP8L2*, and *TNFAIP8L3*) that is represented in the current human genome (Fig 8).

## Discussion

The vertebrate *TNFAIP8* gene family originated from two rounds of whole genome duplication that generated tetra-paralagons containing the *TNFAIP8*, *TNFAIP8L1*, *TNFAIP8L2*, and *TNFAIP8L3* genes (Fig 1). Our analysis indicates that after its divergence from its common ancestor with the spotted gar, the *tnfaip8* ohnolog was lost from the lineage leading to ostariophysi (including zebrafish) and percomorphs (including stickleback) teleosts. This loss could have resulted after the divergence with the spotted gar but before the VGD because no *tnfaip8* orthologs have been identified in other representative teleost species, including stickleback and zebrafish. It is also possible that the *tnfaip8*-containing region duplicated with the TGD to produce, *tnfaip8a* and *tnfaip8b* ohnologs and that two independent events—one associated with an inversion break point and the other associated with the loss of several adjacent genes—led to the loss of both *tnfaip8* TGD ohnologs. Although the two-loss scenario seems less likely by strict parsimony, biologically it seems to be more likely. To help answer this question, we attempted to identify *tnfaip8* gene family members in the genomes of the Japanese and European eels (*Anguilla japonica* and *Anguilla anguilla*, respectively), which are representatives of the Anguilliformes, a basally diverging lineage that arose immediately after the TGD [46]. We performed a TBLASTN query using human and gar TNFAIP8, TNFAIP8L1, TNFAIP8L2, and TNFAIP8L3 protein sequences against the Japanese and European eel genome data available in NCBI but failed to identify any sequences with significant similarity to any of these four genes. It is most likely that the current assemblies are not complete. This question may be addressed later when more sequence information becomes available.

Teleosts like stickleback and zebrafish possess single orthologs of the human *TNFAIP8L1* and *TNFAIP8L3* genes (Figs 2 and 4) while lacking an ortholog of the human *TNFAIP8* gene (Fig 5). It is likely that following the TGD, two co-orthologs to human *TNFAIP8L1* (*tnfaip8l1a* and *tnfaip8l1b*) and *TNFAIP8L3* (*tnfaip8l3a* and *tnfaip8l3b*) were present in the common teleost ancestor genome and that one of the co-orthologs for each gene was subsequently lost, leading to the current condition in teleost genomes. The loss of *tnfaip8* may reflect a non-essential function and/or functional redundancy among *tnfaip8* family members. In contrast, two co-orthologs to the human *TNFAIP8L2* gene remain present in teleosts (*tnfaip8l2a* and *tnfaip8l2b*) (Fig 3). Based upon conserved synteny, it is likely that these co-orthologs arose as a consequence of the TGD and have been retained in the teleost genome. It is likely that the functions of these two TGD ohnologs share between them functions possessed by the single human ortholog *TNFAIP8L2*, as would be expected by sub-functionalization [47]. It is also possible that one or both of these genes or the other teleost *tnfaip8-*family genes have preserved aspects of the missing *TNFAIP8* ortholog’s function [48]. Going forward, it will be imperative to investigate the gene functions of all teleost *tnfaip8* family members so that they could potentially be applied to questions related to human *TNFAIP8* and *TNFAIP8L2* function.

Our findings support the importance of characterizing vertebrate gene histories in developing teleost models for human biomedicine [28, 48]. Teleosts like the zebrafish have been invaluable in understanding embryogenesis and vertebrate development and have been developed as models for host-pathogen interactions [49–65] and tumorigenesis [66–77]. Indeed, through application of gene history analyses, we have previously been able to identify differences in Toll-like receptor (TLR) signaling pathways between zebrafish and humans that are critical to the use and interpretation of the zebrafish as a model for TLR4 (and TICAM2/TRAM adaptor protein) signaling pathways [78, 79]. In the current study, we used the spotted gar genome to help understand the loss of the *tnfaip8* gene from the teleost lineage and described a genome inversion mechanism by which it likely occurred. Our findings highlight the value of the spotted gar as an orthology bridge that can be used to not only identify gene orthologs shared between mammalian and teleost genomes but also to resolve discrepancies related to ohnolog loss. These sorts of studies have broad applicability and will serve to strengthen rationales for the application of teleost models to study problems in human health and disease.

## Supporting information

S1 Fig Amino acid alignment and percent amino acid identity. (A) Clustal Omega alignment of human TNFAIP8, TNFAIP8L1, TNFAIP8L2, and TNFAIP8L3 protein sequences. (B) Percent amino acid identity based on pairwise comparisons between each of the TNFAIP8 family members.
(TIF)

S2 Fig Clustal Omega multiple sequence alignment of the vertebrate TNFAIP8 protein family. (TIF)
