# Supplementary figures and images for: Evolutionary divergence of the vertebrate TNFAIP8 gene family: Applying the spotted gar orthology bridge to understand ohnolog loss in teleosts
Source: PLoS One. 2017 Jun 28;12(6):e0179517. doi: 10.1371/journal.pone.0179517 (PMC5489176; doi:10.1371/journal.pone.0179517)

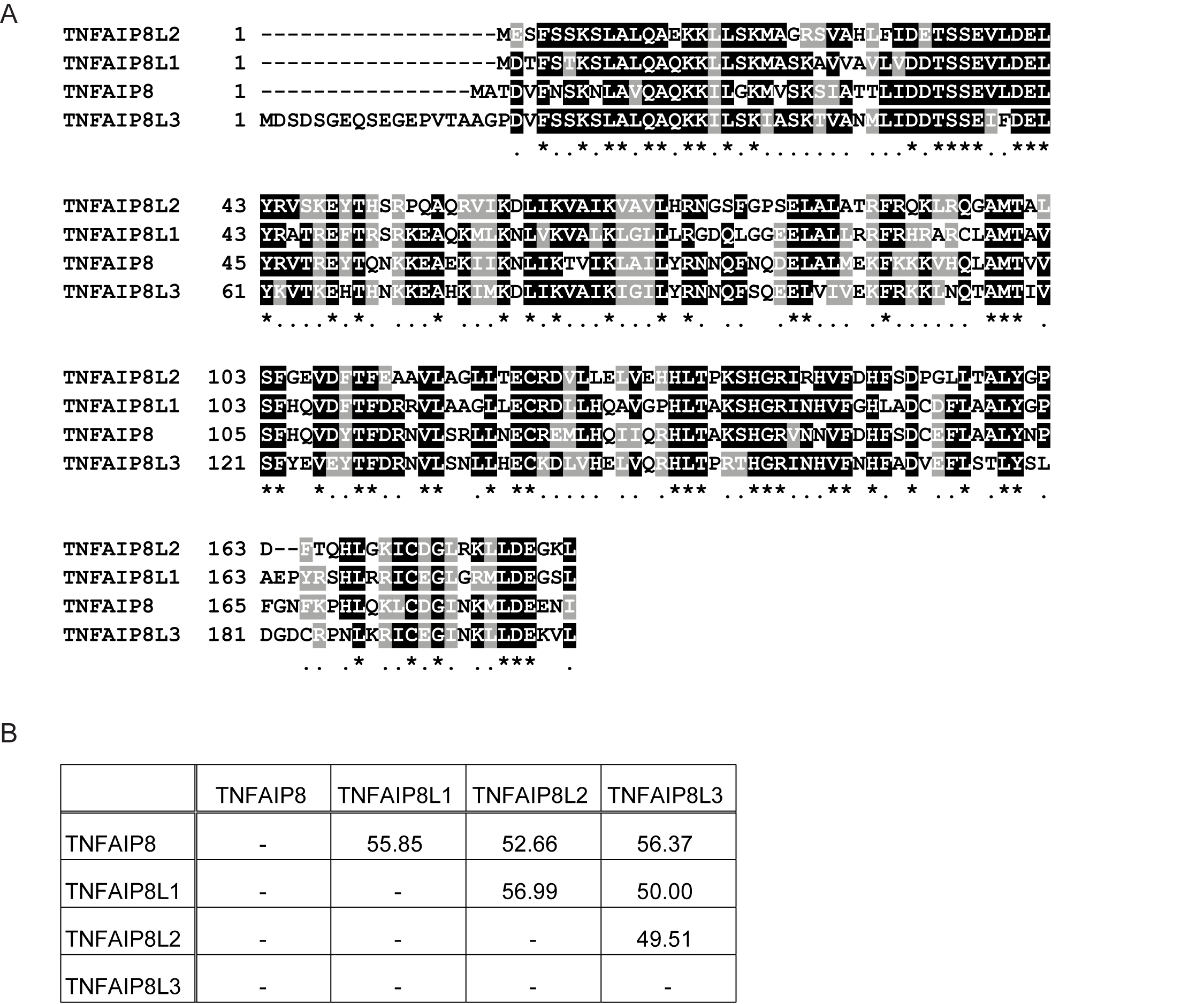

Supplement: S1 Fig — (A) Clustal Omega alignment of human TNFAIP8, TNFAIP8L1, TNFAIP8L2, and TNFAIP8L3 protein sequences. (B) Percent amino acid identity based on pairwise comparisons between each of the TNFAIP8 family members. (TIF) [file pone.0179517.s001.tif]

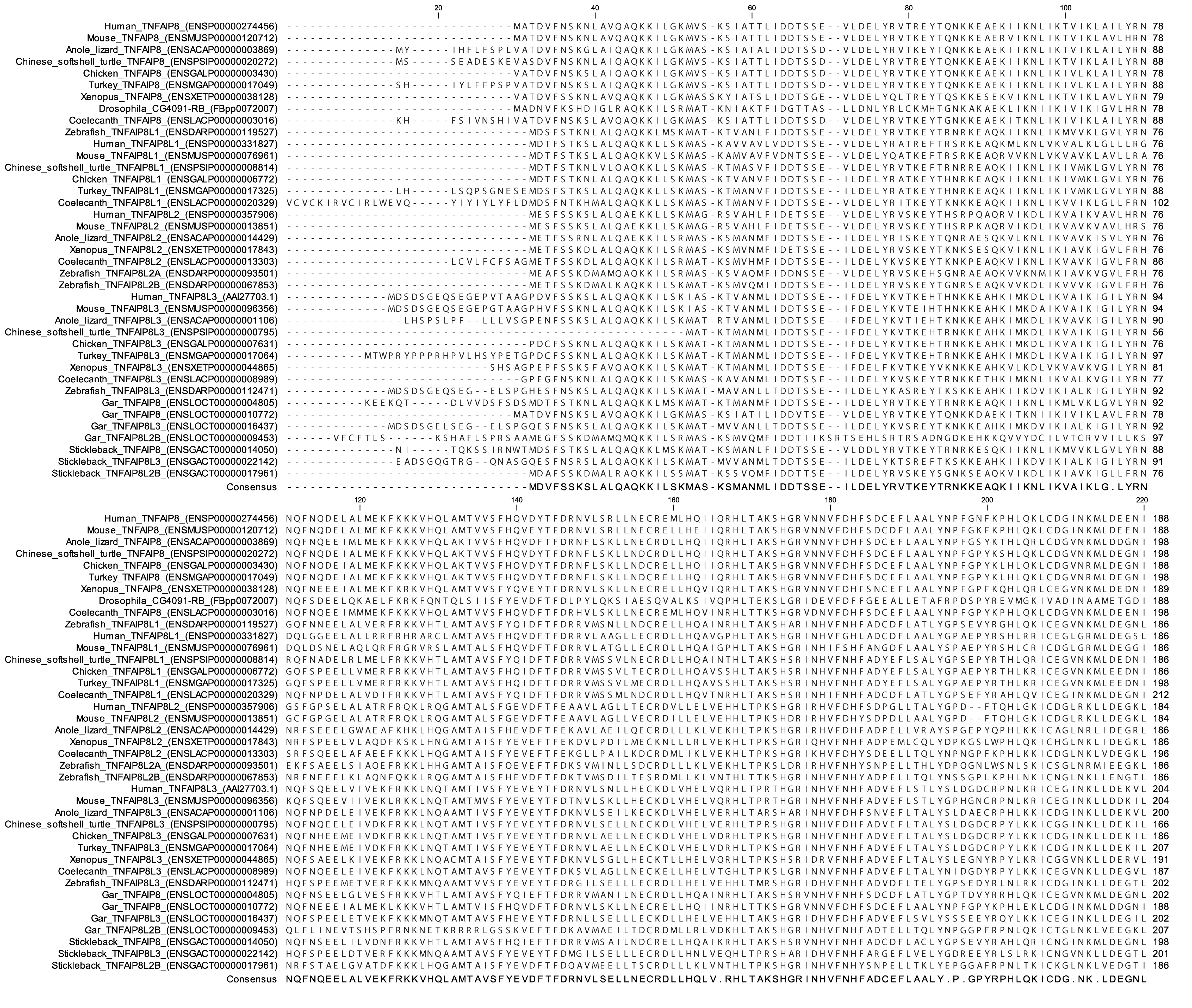

Supplement: S2 Fig — (TIF) [file pone.0179517.s002.tif]
